# Morpho-phylogenetic evidence reveals novel species (Ascomycota) and new records from the Kunming World Horticultural Expo Garden in Yunnan, China

**DOI:** 10.3897/mycokeys.131.185896

**Published:** 2026-04-17

**Authors:** Wenli Li, Rajesh Jeewon, Guilan Zhang, Huan Luo, Liting Zhao, Zhiqin Wang, Changlin Zhao, Hongmin Zhou

**Affiliations:** 1 College of Biological and Food Engineering, Southwest Forestry University, Kunming, 650000, China School of Pharmacy, Zunyi Medical University Zunyi China; 2 Modern Industry School of Edible-Fungi, Southwest Forestry University, Kunming, 650000, China Kunming Institute of Botany, Chinese Academy of Sciences Kunming China; 3 Forest Resources Exploitation and Utilization Engineering Research Center for Grand Health of Yunnan Provincial Universities, Southwest Forestry University, Kunming, 650000, China College of Science, King Saud University Riyadh Saudi Arabia; 4 Department of Health Sciences, Faculty of Medicine and Health Sciences, University of Mauritius, Reduit, 80837, Mauritius Modern Industry School of Edible-Fungi, Southwest Forestry University Kunming China; 5 Department of Zoology, College of Science, King Saud University, Riyadh P.O. Box 2455, Saudi Arabia Forest Resources Exploitation and Utilization Engineering Research Center for Grand Health of Yunnan Provincial Universities, Southwest Forestry University Kunming China; 6 School of Pharmacy, Zunyi Medical University, Zunyi, Guizhou Province 563000, China College of Biological and Food Engineering, Southwest Forestry University Kunming China; 7 Honghe Center for Mountain Futures, Kunming Institute of Botany, Chinese Academy of Sciences, Kunming, Honghe County 654400, Yunnan, China College of Forestry, Southwest Forestry University Kunming China; 8 College of Forestry, Southwest Forestry University, Kunming, 650000, China Faculty of Medicine and Health Sciences, University of Mauritius Reduit Mauritius

**Keywords:** Four taxa, fungi, human-modified landscapes, taxonomy

## Abstract

Fungi represent a kingdom of life with vast, undiscovered diversity. In the unique man-made ecosystem of the Kunming World Horticultural Expo Garden, a site with remarkable plant diversity, four fungal strains were isolated from diseased branches of two widely cultivated plants, *Olea europaea* (olive) and Nandina domestica. Through a comprehensive analysis integrating morphological characteristics and multi-locus phylogenetic data (including LSU, SSU, ITS, *rpb2*, *tef1-α*, and *tub2* sequences), we identified two new species: *Oedohysterium oleae* and *Melomastia kunmingensis*, both of which are phylogenetically and morphologically distinct from known species. Furthermore, we report *M. kevinhydei* as a new host record on *O. europaea*, suggesting a broader host range for this species, and, for the first time, document the sexual morph of the type species *Parapyrenochaeta protearum*, providing crucial morphological insights for this genus, previously known only in its asexual state. These findings underscore the immense value of unique horticultural environments as reservoirs of fungal diversity.

## Introduction

Fungi are a diverse and functionally indispensable component of terrestrial ecosystems, playing central roles in nutrient cycling, plant health, and soil formation (Bajpai et al. 2019). Despite their recognized importance, knowledge of both fungal diversity and ecology remains limited, particularly in human-modified landscapes (Newbound et al. 2010; Mennicken et al. 2020). Accelerated urbanization, which fragments natural habitats into mosaics of built environments and managed green spaces, poses a threat to native fungal communities, yet can also foster novel assemblages (Radeloff et al. 2005). The study of fungal diversity in managed ecosystems, such as urban and ornamental gardens, is therefore gaining attention. These environments harbor unique microbial communities shaped by anthropogenic selection, horticultural practices, and the integration of native and non-native plants, representing underexplored reservoirs for potentially novel fungi.

To examine fungal diversity in such settings, two representative ornamental hosts, *Olea europaea* (olive) and Nandina domestica, were selected. Both are widely cultivated in managed landscapes worldwide. *Olea europaea* is an economically and culturally important species, often cultivated outside its native range, and is a known host to various foliar and wood-inhabiting fungi, including pathogens such as *Colletotrichum* spp. (Sergeeva et al. 2008; Materatski et al. 2018) and *Spilocaea oleaginea* (Lanza et al. 2017). In contrast, Nandina domestica is a commonly planted shrub whose associated mycobiota is poorly characterized, despite occasional reports of foliar diseases like anthracnose (Li et al. 2018; Wang et al. 2018). Comparing the fungal communities of these two hosts allows for the assessment of structural differences between exotic and commonly planted shrubs, facilitating the identification of taxa potentially unique to ornamental garden ecosystems.

Several understudied fungal genera that occur in such environments exemplify the ecological and taxonomic insights that can be gained. The genus *Melomastia* is characterized by a wide ecological distribution, occurring in terrestrial forests (Norphanphoun et al. 2017; Phukhamsakda et al. 2020; Li et al. 2022; Kularathnage et al. 2023), mangroves (Hyde 1992; Hyde et al. 2013; Dayarathne et al. 2020), and freshwater habitats (Hyde 1992). It exhibits a broad host range, colonizing both woody dicotyledonous (Hyde et al. 2018) and some herbaceous plants (Kang et al. 1999; Mapook et al. 2020), yet shows notably high species diversity on specific hosts such as *Olea europaea* (Li et al. 2022). In contrast, the genus *Oedohysterium* is species-poor, with only three formally described members (*O. insidens*, *O. sinense*, and *O. pulchrum*) (https://www.speciesfungorum.org/names/names.asp, 12 March 2026). These species are obligate or specialized saprobes, colonizing dead or senescent woody plant parts, and have a wide geographic distribution across the Americas, Eurasia, and Africa. However, reports from China remain scarce (Boehm et al. 2009). Another relevant genus, *Parapyrenochaeta*, is exclusively asexual and morphologically similar to phoma-like fungi. Its members are saprobic, occur intercontinentally (Australia, Africa, Europe), and are typically found on leaves or needles of specific woody hosts such as *Acacia*, *Protea*, and *Pinus* (Crous et al. 2011; Crous et al. 2016; Valenzuela-Lopez et al. 2018). Together, these genera illustrate varied ecological strategies, host associations, and distribution patterns that underscore the underexplored fungal diversity in managed garden ecosystems.

The Kunming World Horticultural Expo Garden, a typical urban landscaped garden, hosts over 2,500 plant varieties (including 112 rare and endangered species), creating a rich and heterogeneous environment for fungal colonization. From this setting, four fungal strains were isolated from olive (*Olea europaea*) and nandina (Nandina domestica). Subsequent comprehensive analysis, integrating both morphological characteristics and molecular phylogenetic data, resulted in the identification of two new species, one new host record, and the first record of the teleomorph of a known species. This study provides taxonomic details on their morphology, identification, and phylogenetic relationship with extant species.

## Materials and methods

### Sample Collection, Specimen Examination, and Fungal Isolation

Fresh fungal materials were collected from diseased branches of *Olea europaea* and Nandina domestica at the World Horticultural Expo Garden, Kunming, in Yunnan Province (25°04'31.8179"N, 102°45'38.0231"E; Alt.: 1960 m). After the important collection details were noted (Rathnayaka et al. 2025), specimens were transported to the mycology laboratory at Southwest Forestry University in a Kraft envelope.

Fungal structures on the host substrates were observed using a Motic SMZ 161 stereomicroscope, and ascomata were photographed using a digital camera mounted on the stereomicroscope. Micro-morphological characteristics were observed and photographed with a Zeiss Axioscope 5 compound microscope fitted with an Axiocam 305 color digital camera. Micro-morphological characteristics were measured by ZEN 3.7 software. Images used for figures were edited with Adobe Photoshop CS6 software (Adobe Systems, United States).

Fungal isolation was performed using single-spore isolation following Senanayake et al. (2020). Germinated spores were individually transferred to potato dextrose agar (PDA) plates under aseptic conditions and incubated at room temperature in daylight for 3–4 weeks.

Dry herbarium materials were preserved at the Herbarium of Cryptogams Kunming Institute of Botany, Academia Sinica (**KUN-HKAS**) and Herbarium of Southwest Forestry University (**SWFC**), Kunming, Yunnan Province, China. Ex-type living cultures were deposited at the China General Microbiological Culture Collection Centre (**CGMCC**) and the Southwest Forestry University Cultural Collection (**SWFCCC**). The new species name was registered to the Faces of Fungi database (https://www.facesoffungi.org/) (Jayasiri et al. 2015) and MycoBank (http://www.MycoBank.org) (Crous et al. 2004). New species were established following the recommendations of Jeewon and Hyde (2016).

### DNA extraction, amplification, and sequencing

Fresh fungal mycelium (500 mg) was scraped from the margin of a PDA plate transferred into a 1.5 mL centrifuge tube, and ground using a hand-held tissue grinder. Genomic DNA was extracted according to the manufacturer’s protocol for the EZ gene^TM^ Fungal gDNA kit (GD2416) (Beijing TsingKe Biotech Co., Ltd., Beijing, China). DNA amplification was performed using the polymerase chain reaction (PCR). The following primers were used for PCR amplification and sequencing: ITS5/ITS4 for the internal transcribed spacer regions (ITS1, 5.8S rDNA and ITS2) (White 1994), LR0R/LR5 for the nuclear ribosomal large subunit 28S rDNA (LSU) (Rehner and Samuels 1995); NS1/NS4 for the nuclear ribosomal small subunit 18S rDNA (SSU) (White 1994), EF1-983F/EF1-2218R for the partial translation elongation factor 1-alpha (*tef1-α*) (Sung et al. 2007), RPB2-5F/RPB2-7cR for the partial RNA polymerase second largest subunit region (*rpb2*) (Liu et al. 1999), and Bt2a/Bt2b for the β-tubulin (*tub2*) (Glass and Donaldson 1995).

The PCR reaction was performed at a volume of 25 µL. PCR mixtures contained 12.5 µL of 2 × Power Taq PCR MasterMix (Bioteke Co., China), 1 µL of each primer (10 µM), 1 µL genomic DNA and 9.5 µL ddH_2_O. PCR reaction parameters for LSU, SSU, ITS, *tub2* and *tef1-α* were as follows: initial denaturation at 94 °C for 3 min, followed by 35 cycles of denaturation at 94 °C for 45 s, annealing at 55 °C for 50 s, extension at 72 °C for 1 min, and a final extension at 72 °C for 10 min. The PCR amplification of *rpb2* was performed as follows: an initial denaturation at 95 °C for 5 min, followed by 40 cycles of denaturation at 95 °C for 1 min, annealing at 52 °C for 2 min, extension at 72 °C for 90 s, and a final extension at 72 °C for 10 min. Purification and sequencing of PCR products were carried out at Beijing Tsingke Biological Engineering Technology and Services Co., Ltd (Beijing, P.R. China). The newly generated sequences were deposited in GenBank.

### Phylogenetic analyses

Newly generated sequences were subjected to BLASTn searches (https://blast. ncbi.nlm.nih.gov), and closely related sequences were downloaded from GenBank to construct datasets for further analyses (Table 1). Each gene dataset was aligned using MAFFT v7 (https://mafft.cbrc.jp/alignment/server/large.html) (Katoh et al. 2019) and improved, when necessary, in BioEdit v. 7.0 (Hall 1999).

**Table 1.** List of the taxa used in the phylogenetic analyses, with GenBank accession numbers.

Taxa names	Strain numbers	LSU	SSU	*tef1*-α	ITS	*rpb2*	*tub2*
*Acrospermum adeanum*	M133	EU940104	EU940031	N/A	N/A	N/A	N/A
*Anisomeridium phaeospermum*	MPN539	JN887394	JN887374	JN887418	N/A	N/A	N/A
*Anisomeridium ubianum*	MPN94	N/A	JN887379	JN887421	N/A	N/A	N/A
*Astragalicola vasilyevae*	MFLUCC 17-0832 T	MG828986	MG829098	MG829193	NR_157504	MG829248	N/A
*Camarosporidiella elongata*	AFTOL-ID 1568	DQ678061	DQ678009	DQ677904	N/A	DQ677957	N/A
*Camarosporidiella eufemiana*	MFLUCC 17-0207 T	MF434233	MF434321	MF434408	MF434145	N/A	N/A
*Gloniopsis calami*	MFLUCC 15-0739 T	NG_059715	NG_063621	KX671965	NR_164398	N/A	N/A
*Gloniopsis kenyensis*	GKM1010 T	GQ221891	N/A	N/A	N/A	N/A	N/A
*Gloniopsis praelonga*	CMW 19983	FJ161193	FJ161152	N/A	N/A	N/A	N/A
*Gloniopsis praelonga*	CBS 112415 T	FJ161173	FJ161134	FJ161090	N/A	N/A	N/A
*Glonium circumserpens*	CBS 123342	FJ161208	N/A	N/A	N/A	N/A	N/A
*Glonium circumserpens*	EB 0332	FJ161200	FJ161160	FJ161108	N/A	N/A	N/A
*Glonium stellatum*	ANM32	GQ221887	N/A	GQ221926	N/A	N/A	N/A
*Glonium stellatum*	CBS 207.34	FJ161179	N/A	N/A	N/A	N/A	N/A
*Hysterium angustatum*	CBS 123334	FJ161207	N/A	N/A	N/A	N/A	N/A
*Hysterium pulicare*	CBS 123377	FJ161201	N/A	N/A	N/A	N/A	N/A
*Hysterium pulicare*	ANM85	GQ221898	N/A	GQ221934	N/A	N/A	N/A
*Hysterium vermiforme*	GKM1234	GQ221897	N/A	GQ221929	N/A	N/A	N/A
*Hysterobrevium baoshanense*	MFLUCC 16-2162	KX772765	KX772767	KX772769	MZ467049	N/A	N/A
*Hysterobrevium constrictum*	JCM 2753 T	NG_078641	N/A	N/A	NR_175063	N/A	N/A
*Hysterobrevium hyde*	MBSZU 25-001	PQ849540	PQ849542	PQ868902	PQ849538	N/A	N/A
*Hysterobrevium mori*	CBS 123335	FJ161202	N/A	N/A	N/A	N/A	N/A
*Hysterobrevium rosae*	MFUCC 14-0551 T	MH535897	N/A	MH535879	N/A	N/A	N/A
*Hysterographium flexuosum*	GKM1262c	GQ221886	N/A	GQ221935	N/A	N/A	N/A
*Hysterographium fraxini*	MFLU 15–3035	MH535900	MH535889	MH535882	N/A	N/A	N/A
*Hysterographium fraxini*	CBS 109.43	FJ161171	FJ161132	FJ161088	N/A	N/A	N/A
*Leptosphaeria conoidea*	CBS 616.75	JF740279	JF740099	N/A	JF740201	KT389639	KT389804
*Leptosphaeria doliolum*	CBS 505.75	NG_068574	NG_062778	GU349069	NR_155309	KY064035	JF740144
Massarina cisti	CBS 266.62	AB807539	AB797249	AB808514	LC014568	FJ795464	
Massarina eburnea	CBS 473.64 T	GU301840	GU296170	GU349040	AF383959	GU371732	GU301840
*Melomastia aquilariae*	ZHKUCC 23-0073 T	OR807856	OR807854	OR832867	N/A	N/A	N/A
*Melomastia beihaiensis*	KUMCC 21-0084 T	MZ726990	MZ727002	N/A	NR_190244	N/A	N/A
*Melomastia chinensis*	GMB6242 T	PQ860472	PQ860573	PQ826966	PQ874026	N/A	N/A
*Melomastia chromolaenae*	MFLUCC 17-1434 T	N/A	MT214413	MT235800	N/A	N/A	N/A
*Melomastia clematidis*	MFLUCC 17-2092 T	MT214607	MT226718	MT394663	MT310651	N/A	N/A
*Melomastia diqingensis*	KUMCC 21-0536 T	OQ170873	OQ168224	OR613413	OQ158951	N/A	N/A
*Melomastia distoseptata*	MFLUCC 21-0102	MT860427	N/A	N/A	MT864349	N/A	N/A
*Melomastia distoseptata*	PUFNI 17640	MH971237	MN582765	N/A	MK024391	N/A	N/A
*Melomastia fulvicomae*	MFLUCC 17-2083 T	MT214608	MT226719	MT394664	MT310652	N/A	N/A
*Melomastia fusispora*	CGMCC 3.20618 T	OK623464	OK623494	OL335189	NR_185653	N/A	N/A
*Melomastia guangdongensis*	GMBCC1046 T	PQ530970	PQ530975	PQ559185	N/A	N/A	N/A
*Melomastia hydei*	MBSZU 25–003 T	PQ844642	PQ844644	N/A	PQ849544	N/A	N/A
*Melomastia italica*	MFLUCC 15-0160 T	MG029458	MG029459	N/A	N/A	N/A	N/A
*Melomastia kevinhydei*	UESTCC 25.0042 T	PQ773421	PQ773437	PV059193	PQ773401	N/A	N/A
***Melomastia kevinhydei***	**SWFCCC 250002**	**PX928702**	**PX928698**	**PX959668**	**PX937321**	N/A	N/A
***Melomastia kunmingensis***	**CGMCC3.29467 T**	**PX928701**	**PX928697**	PX959667	**PX937320**	N/A	N/A
*Melomastia loropetalicola*	ZHKUCC 22-0174 T	OQ379412	OQ379411	N/A	N/A	N/A	N/A
*Melomastia maolanensis*	GZCC 16-0102 T	KY111905	KY111906	KY814762	N/A	N/A	N/A
*Melomastia maomingensis*	ZHKUCC 23-0038 T	PP809724	PP809704	PP812255	OR825372	N/A	N/A
*Melomastia neothailandica*	MFLU 17-2589 T	NG_068294	N/A	N/A	N/A	N/A	N/A
*Melomastia oleae*	CGMCC 3.20619 T	OK623466	OK623496	OL335191	NR_185654	N/A	N/A
*Melomastia phetchaburiensis*	MFLUCC 15-0951 T	MF615402	MF615403	N/A	N/A	N/A	N/A
*Melomastia puerensis*	ZHKUCC 23-0802 T	OR922309	OR922340	OR966284	OR941077	N/A	N/A
*Melomastia pyriformis*	ZHKUCC 22-0175 T	OP791870	OP739334	OQ718392	N/A	N/A	N/A
*Melomastia rhizophorae*	JK 5456 A	GU479799	N/A	GU479860	N/A	N/A	N/A
*Melomastia septata*	MFLUCC 22-0112 T	NG_242029	N/A	OP760198	NR_189402	N/A	N/A
*Melomastia sichuanensis*	CGMCC 3.20620 T	OK623469	OK623500	OL335195	N/A	N/A	N/A
*Melomastia sinensis*	MFLUCC 17-1344 T	MG836699	MG836700	N/A	N/A	N/A	N/A
*Melomastia thailandica*	MFLU 17-2610 T	MN017858	MN017923	MN077069	N/A	N/A	N/A
*Melomastia thamplaensis*	MFLUCC 15-0635 T	KX925435	KX925436	KY814763	N/A	N/A	N/A
*Melomastia tiomanensis*	MFLUCC 13-0440 T	KC692156	KC692155	KC692157	N/A	N/A	N/A
*Melomastia winteri*	CGMCC 3.20621 T	OK623471	OK623502	OL335197	NR_185655	N/A	N/A
*Melomastia yunnanensis*	GMBCC1009 T	PQ530973	PQ530978	PQ559188	N/A	N/A	N/A
*Muyocopron dipterocarpi*	MFLU 17.2608	KU726966	KU726969	MT136754	N/A	N/A	N/A
*Muyocopron garethjonesii*	MFLUCC 16-2664 T	KY070274	KY070275	N/A	N/A	N/A	N/A
*Muyocopron lithocarpi*	MFLUCC 14-1106 T	KU726967	KU726970	MT136755	MT137786	N/A	N/A
*Mytilinidion resinicola*	CBS 304.34	MH867038	N/A	FJ161101	MH855535	N/A	N/A
*Mytilinidion scolecosporum*	CBS 305.34	MH867039	NG_016510	FJ161102	MH855536	N/A	N/A
*Neocucurbitaria ribicola*	CBS 142394 T	MF795785	MF795840	MF795873	MF795785	MF795827	MF795911
*Neoleptosphaeria jonesii*	MFLUCC 16-1442	KY211870	NG_063625	KY211872	NR_152375	N/A	N/A
*Neophaeosphaeria agaves*	CBS 136429 T	CBS 136429	KF777227	N/A	N/A	NR_137833	N/A
*Neophaeosphaeria filamentosa*	CBS 102202 T	GQ387577	GQ387516	N/A	JF740259	GU371773	N/A
*Neophaeosphaeria phragmiticola*	KUMCC 16-0216 T	MG837009	MG837008	MG838020	N/A	N/A	N/A
*Neopyrenochaeta acicola*	CBS 812.95 T	GQ387602	NG_065567	N/A	NR_160055	LT623271	LT623232
*Neopyrenochaeta cercidis*	MFLU 18-2089	MK347932	NG_065769	N/A	MK347718	MK434908	N/A
*Neopyrenochaeta fragariae*	CBS 101634 T	GQ387603	GQ387542	N/A	LT623217	LT623270	LT623231
*Neopyrenochaeta inflorescentiae*	CBS 119222 T	EU552153			EU552153	LT623272	LT623233
*Neopyrenochaeta maesuayensis*	MFLUCC 14-0043	MT183504	N/A	MT454042	NR_170043	N/A	N/A
*Neopyrenochaeta telephoni*	CBS 139022 T	NG_067485	N/A	N/A	KM516291	LT717685	LT717678
*Oedohysterium insidens*	ANM1443	GQ221882	N/A	N/A	N/A	N/A	N/A
*Oedohysterium insidens*	CBS 238.34 T	MH866997	FJ161142	FJ161097	N/A	N/A	N/A
***Oedohysterium oleae***	**CGMCC3.29466 T**	**PX928703**	**PX928699**	**PZ124612**	**PZ191797**	N/A	N/A
*Oedohysterium sinense*	EB 0339	GU397348	GU397364	GU397339	N/A	N/A	N/A
*Oedohysterium sinense*	CBS 123345	FJ161209	NG016513	N/A	N/A	N/A	N/A
*Parapyrenochaeta acaciae*	CBS 141291T	NG_070398	N/A	N/A	KX228265	LT717686	LT717679
*Parapyrenochaeta maryellenpeartiae*	BRIP 74437a T	N/A	N/A	N/A	OQ297061	N/A	N/A
***Parapyrenochaeta protearum***	**SWFCCC 250004**	**PX928704**	**PX928700**	**PX959669**	**PX937322**	N/A	N/A
*Parapyrenochaeta protearum*	CBS 131315 T	JQ044453	N/A	N/A	JQ044434	LT717683	LT717677
*Parapyrenochaeta protearum*	CBS 137997	KJ869209	N/A	N/A	KJ869152	LT717684	KJ869249
*Pseudopyrenochaeta lycopersici*	CBS 306.65 T	EU754205	N/A	N/A	NR_103581	LT717680	NR_103581
*Pseudopyrenochaeta terrestris*	CBS 282.72 T	LT623216	N/A	N/A	LT623228	LT623287	LT623246
*Psiloglonium araucanum*	CBS 112412	FJ161172	FJ161133	FJ161089	N/A	N/A	N/A
*Psiloglonium colihuae*	MFLUCC 11-0178	KP744511	N/A	N/A	KP744466	N/A	N/A
*Psiloglonium multiseptatum*	MFLUCC 11-0200	KP744512	N/A	N/A	N/A	N/A	N/A
*Psiloglonium sasicola*	MFLUCC 10-0565	KP744513	N/A	N/A	KP744467	N/A	N/A
*Psiloglonium simulans*	CBS 206 34	MH866971	FJ161139	FJ161094	N/A	N/A	N/A
*Psiloglonium simulans*	ANM1557	GQ221873	N/A	N/A	N/A	N/A	N/A
*Pyrenochaeta fraxinina*	43E	N/A	N/A	N/A	MT547827	OM806005	MT547862
*Pyrenochaeta gentianicola*	MAFF 425530	N/A	N/A	N/A	AB499789	N/A	AB500024
*Pyrenochaeta hakeae*	CPC 28920	NG_059751	N/A	N/A	NR_155692	KY173593	KY173613
*Pyrenochaeta nobilis*	CBS 407.76 T	EU754206	DQ898287	DQ677936	NR_103598	DQ677991	MF795916
*Pyrenochaeta pinicola*	CBS 137997	KJ869209	N/A	N/A	KJ869152	LT717684	KJ869249
*Pyrenochaetopsis botulispora*	CBS 142458 T	LN907440	N/A	N/A	LT592945	LT593084	LT593014
*Pyrenochaetopsis globosa*	CBS 143034 T	LN907418	N/A	N/A	LT592934	LT593072	LT593003
*Pyrenochaetopsis paucisetosa*	CBS 142460 T	LN907336	N/A	N/A	LT592897	LT593035	LT592966
*Pyrenochaetopsis setosissima*	CBS 119739 T	GQ387632	N/A	N/A	LT623227	LT623285	LT623245
*Quixadomyces cearensis*	HUEFS 238438 T	NG_066409	N/A	N/A	NR_160606	N/A	N/A
*Quixadomyces hongheensis*	HKAS112347	MW541823	MW541834	MW556135	MW541827	N/A	MW556138
*Quixadomyces hongheensis*	KUMCC 20-0215 T	MW264194	MW264224	MW256816	NR_172441	MW269529	MW256804
*Quixadomyces hongheensis*	HKAS112346	MW541822	MW541833	MW556134	MW541826	MW556136	MW556137
*Quixadomyces sanctacrucensis*	CBS 151613	PP457952	N/A	PP461448	PP457806	PP461447	N/A
*Rhytidhysteron bruguierae*	MFLU 18-0571 T	MN017833	MN017901	MN077056	N/A	N/A	N/A
*Rhytidhysteron camporesii*	KUN-HKAS 104277 T	MN429072	N/A	MN442087	MN429069	N/A	N/A
*Rhytidhysteron chromolaenae*	MFLUCC 17-1516 T	MN632456	MN632467	MN635663	MN632461	N/A	N/A
*Rhytidhysteron coffeae*	KUMCC 21-0489 T	OP526406	OP526412	OP572201	OP605963	N/A	N/A
*Rhytidhysteron erioi*	MFLU 16-0584 T	MN429071	N/A	MN442086	MN429068	N/A	N/A
*Rhytidhysteron mengziense*	KUMCC 21-0490 T	OP526396	OP526414	OP572203	OP526402	N/A	N/A
*Rhytidhysteron mexicanum*	RV17107.1 T	MT626028	N/A	N/A	MT626026	N/A	N/A
*Rhytidhysteron bannaense*	KUMCC 21-0482 T	OP526408	OP526395	OP572199	OP526398	N/A	N/A
*Rhytidhysteron magnoliae*	MFLUCC 18-0719 T	MN989384	MN989382	MN997309	MN989383	N/A	N/A
*Rhytidhysteron mangrovei*	MFLU 18-1894 T	MK357777	N/A	MK450030	MK425188	N/A	N/A
*Rhytidhysteron neorufulum*	MFLUCC 13-0216 T	KU377566	KU377571	KU510400	KU377561	N/A	N/A
*Rhytidhysteron rufulum*	MFLUCC 14-0577 T	KU377565	KU377570	KU510399	KU377560	N/A	N/A
*Rhytidhysteron tectonae*	MFLUCC 13-0710 T	KU764698	KU712457	KU872760	KU144936	N/A	N/A
*Rhytidhysteron thailandicum*	MFLUCC 14-0503 T	KU377564	KU377569	KU497490	KU377559	N/A	N/A
*Rhytidhysteron xiaokongense*	KUMCC 20-0160 T	MZ346012	MZ346017	MZ356246	MZ346022	N/A	N/A
*Rhytidhysteron yunnanense*	KUMCC 21-0485 T	OP526404	OP526400	OP572205	OP526410	N/A	N/A
*Seltsamia ulmi*	CBS 143002 T	MF795794	MF795794	MF795882	MF795794	MF795836	MF795918
*Staurosphaeria lycii*	MFLUCC 17-0210 T	MF434284	MF434372	MF434458	MF434196	N/A	N/A
*Staurosphaeria lycii*	MFLUCC 17-0211	MF434285	MF434373	MF434459	MF434197	N/A	N/A
*Stigmatodiscus labiatus*	CBS 144700 T	MH756065	MH756065	MH756083	MH756065	N/A	N/A
*Stigmatodiscus oculatus*	CBS 144701 T	MH756069	N/A	MH756086	NR_164035	N/A	N/A
*Stigmatodiscus pruni*	CBS 142598 T	KX611110	KX611110	KX611111	KX611110	N/A	N/A
*Xenopyrenochaetopsis pratorum*	CBS 445.81 T	NG_057858	NG_062792	N/A	NR_111623	KT389671	KT389846

* Remarks: The superscript T denotes ex-type isolates. “N/A” denotes the sequence is unavailable. The newly generated sequences and new species are indicated in black bold font.

Phylogenetic trees were inferred using both Maximum Likelihood (ML) and Bayesian Inference (BI) methods. The ML analysis was conducted on the CIPRES web portal using RAxML-HPC2 on XSEDE (v 8.2.8) under the GTR+G+I model of sequence evolution (Miller et al. 2010). A total of 1000 bootstrap replicates were performed to assess branch support. The BI tree was performed with MrBayes on XSEDE (3.2.7a) (Ronquist et al. 2012) by the Markov Chain Monte Carlo (MCMC) method to evaluate Bayesian posterior probabilities (BYPP). The best-fit nucleotide substitution model parameters were selected independently for different gene regions under the Akaike information criterion (AIC) implemented in PAUP v. 4.0b10 (Table 2). The alignment was run for 2,000,000 generations with two independent runs, each containing four Markov Chains Monte Carlo (MCMC) chains and sampling every 100 iterations. The initial 10% of sampled trees were discarded as “burn-in”, and the remaining trees were used to calculate the posterior probability (PP).

**Table 2.** Maximum-likelihood (ML) and Bayesian (BI) analyses results for each sequenced dataset.

Analyses		Fig. 1	Fig. 2	Fig. 3
Number of taxa		40	48	42
Gene regions		LSU, SSU, ITS, *tef1-α*	LSU, SSU, ITS, *tef1-α*	LSU, SSU, ITS, *tef1-α*, *rpb2*, *tub2*
Number of character positions (including gaps)		3459	3443	6910
ML optimization likelihood value		-18454.19413	-17883.66811	-34623.42984
Distinct alignment patterns in the matrix		1346	1213	2091
Number of undetermined characters or gaps (%)		25.99%	32.75%	50.49%
Estimated base frequencies	A	0.233069	0.242905	0.246881
	C	0.270376	0.247368	0.245554
	G	0.291921	0.276703	0.265653
	T	0.204634	0.233025	0.241912
Substitution rates	AC	1.160746	1.233243	1.573791
	AG	2.658031	2.782142	4.198585
	AT	1.197577	1.070004	1.853201
	CG	1.057219	0.824492	1.095599
	CT	7.771736	7.270692	8.145483
	GT	1	1	1
Proportion of invariable sites (I)		0.434734	0.509392	0.487692
Gamma distribution shape parameter (α)		0.58757	0.572768	0.440792
Final split frequency		0.009638	0.009921	0.009494
The total of unique site patterns		1344	1217	2094
BI (model of each gene region)	LSU	GTR+I+G	GTR+I+G	GTR+I+G
	SSU	SYM+I+G	GTR+I+G	HKY+I
	ITS	GTR+G	SYM+I+G	GTR+I+G
	*tef1-α*	GTR+I+G	GTR+I+G	GTR+I+G
	*rpb2*	–	–	GTR+I+G
	*tub2*	–	–	HKY+G

The resulting trees were viewed in FigTree v.1.4.4 (Rambaut 2016) and the final layout was done with Adobe Illustrator CS5 (Adobe Systems, San Jose, CA, USA).

### Genealogical Concordance Phylogenetic Species Recognition (GCPSR) analysis

Detection of recombination events was performed using the Pairwise Homoplasy Index (PHI or Φw) test (Bruen et al. 2006) implemented in SplitsTree4 software (Huson 1998; Huson and Bryant 2006; Huson and Bryant 2024), based on the Genealogical Concordance Phylogenetic Species Recognition (GCPSR) model (Quaedvlieg et al. 2014). The analysis utilized a concatenated nucleotide sequence alignment of loci from phylogenetically related strains. The core principle of the method relies on identifying refined incompatibility between pairs of aligned sites, which reflects whether the genealogical history of two nucleotide positions can be parsimoniously explained without invoking recurrent or convergent mutations (homoplasies). A statistically significant level of site incompatibility suggests the action of recombination rather than homoplasy. Phylogenetic relationships were visualized using a split graph generated with LogDet transformation and the splits decomposition algorithm. Statistical significance was determined using a Φw threshold of 0.05; values below this cutoff indicate significant evidence of recombination in the dataset.

## Results

### Phylogenetic analysis

**Figure 1.**
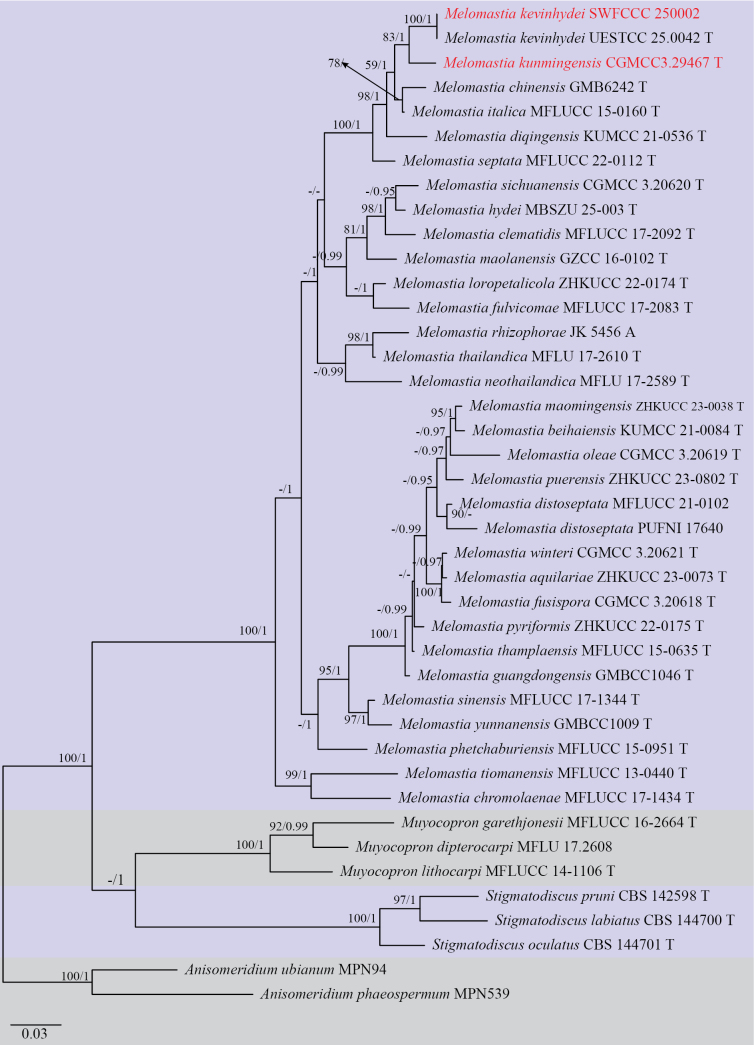
Phylogenetic tree generated from maximum likelihood analysis based on a combined dataset of LSU, SSU, ITS, and *tef1-α* sequences. Bootstrap support values for ML (≥75%) and Bayesian posterior probabilities (≥0.95) are given at the nodes (ML/BYPP). Ex-type strains are indicated in T, and the new strains are shown in red. The tree is rooted with *Anisomeridium ubianum* MPN94 and *A. phaeospermum* MPN539.

**Figure 2.**
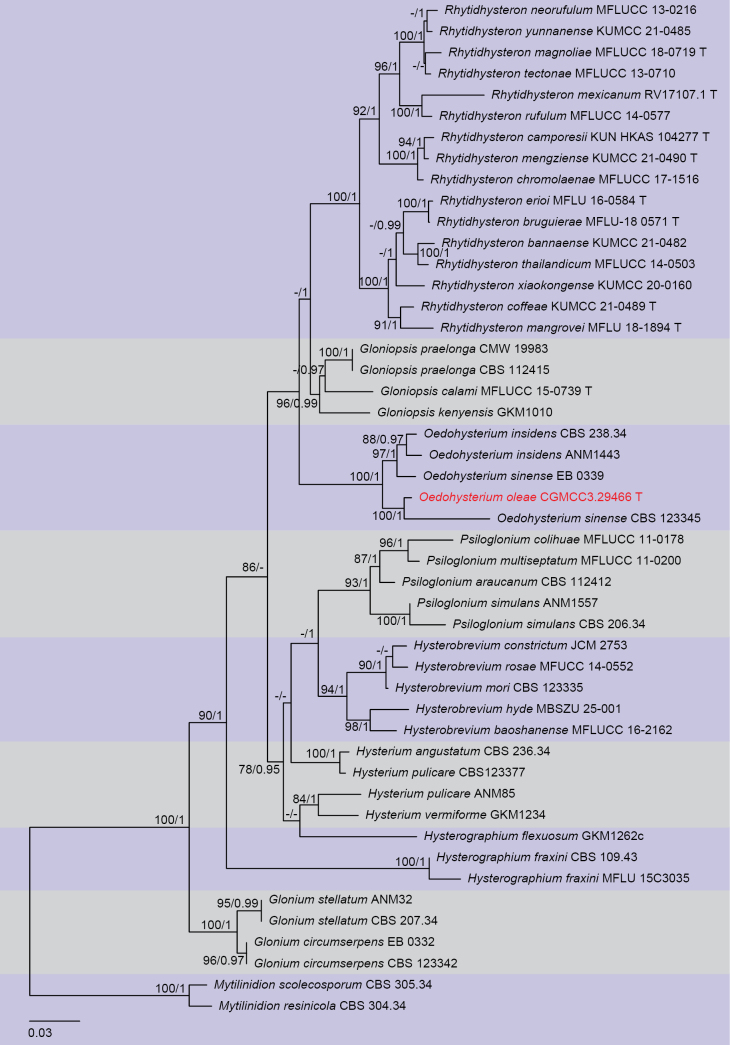
Phylogenetic tree generated from maximum likelihood analysis based on a combined dataset of LSU, SSU, *tef1-α*, and ITS sequences. Bootstrap support values for ML (≥75%) and Bayesian posterior probabilities (≥0.95) are given at the nodes (ML/BYPP). Ex-type strains are indicated in T, and the new strains are shown in red. The tree is rooted with *Mytilinidion scolecosporum* CBS 305.34 and *M. resinicola* CBS 304.34.

**Figure 3.**
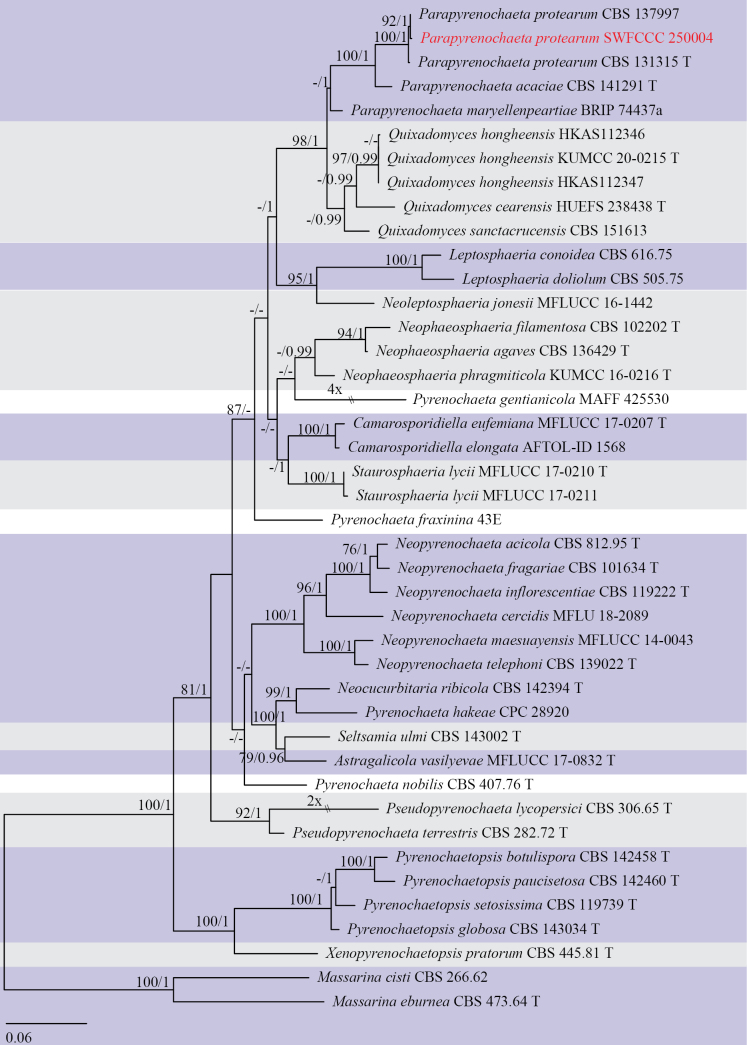
Phylogenetic tree generated from maximum likelihood analysis based on a combined dataset of SSU, LSU, ITS, *rpb2*, *tef1-α*, and *tub*2 sequences. Bootstrap support values for ML (≥75%) and Bayesian posterior probabilities (≥0.95) are given at the nodes (ML/BYPP). Ex-type strains are indicated in T, and the new strains are shown in red. The tree is rooted with Massarina cisti CBS 266.62 and *M. eburnean* CBS 473.64.

Three analyses were performed in this study. The first is a phylogenetic overview of the genera treated in Pleurotremataceae (Fig. 1); the second analysis focuses on Hysteriaceae (Fig. 2); while the third dataset represents the genera in the pyrenochaeta-like group (Fig. 3). Other details related to both ML and BI analyses from Hysteriaceae, Pleurotremataceae, and the pyrenochaeta-like group datasets are presented in Table 2. Phylogenetic analyses using both Maximum Likelihood (ML) and Bayesian Inference (BI) methods produced trees with congruent topologies.

In the phylogenetic tree, our four isolates obtained in the present study were resolved into three distinct family-level clades within Pleosporales. These isolates represent two new phylogenetic species and two known species. *Melomastia kunmingensis* formed a distinct clade and is closely related to *M. kevinhydei* with 83 ML and 1.00 BYPP support values; the new collection SWFCCC 250002 clustered as a sister taxon to *M. kevinhydei* (UESTCC 25.0042) with 100 ML and 1.00 BYPP statistical support (Fig. 1). *Oedohysterium oleae* grouped with the *O. sinense* (CBS 123345) with 100 ML and 1.00 BYPP support values (Fig. 2). The new collection SWFCCC 250004 constituted a well-supported clade as a sister taxon to *Parapyrenochaeta protearum* (CBS 131315 and CBS 137997) (Fig. 3).

#### Melomastia kunmingensis

Taxon classificationFungiSordariomycetes

W.L. Li, & C.L. Zhao sp. nov.

94C6746E-88AF-549E-96CE-378590EFCDA7

MycoBank No: 862011

Facesoffungi Number: FoF19140

Fig. 4

##### Etymology.

Named after the type locality, “Kunming World Horticultural Expo Garden”.

**Figure 4.**
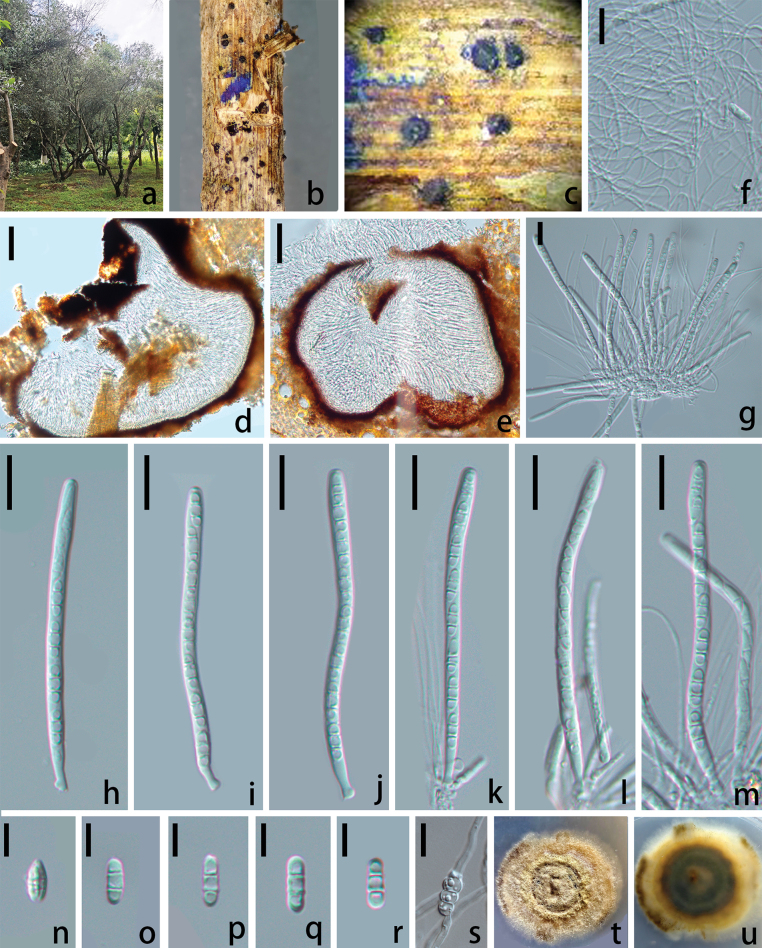
*Melomastia kunmingensis* sp. nov., (HKAS 151735). **a**. Habitat; **b, c**. Ascomata on the host; **d, e**. Vertical section of ascoma; **f**. Hamathecium; **g–m**. Asci; **n–r**. Ascospores; **s**. Germinated ascospore; **t, u**. Culture (**t**. From above; **u**. From below). Scale bars: 50 μm (**d, e**); 20 μm (**f–m**); 10 μm (**n–s**).

##### Holotype.

HKAS 151735.

##### Description.

***Saprobic*** on diseased branches of *Olea europaea* L. **Sexual morph: *Ascomata*** visible as raised, dome-shaped, black dots on the host surface, solitary, scattered to gregarious, in vertical section 225–280 µm high × 285–360 µm diam. (x̄ = 252 × 322.5 µm, n = 10), obpyriform, semi-immersed to erumpent, black, carbonaceous, ostiolate. ***Ostiolar*** 50–75 µm wide (n = 5), central, black, conical, carbonaceous, internally lined with hyaline periphyses. ***Peridium*** 18–22 µm wide (x̄ = 20 µm, n = 10), comprising dense, several layers of brown to dark brown, compressed cells of ***textura angularis*** to ***textura prismatica***. ***Hamathecium*** 2–3 µm wide, numerous, filamentous, flexuose, branched, hyaline, and embedded in a gelatinous matrix. ***Asci*** 107–121 × 6–7 µm (x̄ = 114 × 6 µm, n = 30), bitunicate, 8-spored, cylindrical, pedicellate, apically rounded, with minor ocular chamber. ***Ascospores*** 5–7 × 16–20 µm (x̄ = 6 × 18 µm, n = 30), uniseriate, hyaline, cylindrical, with rounded ends, 2-septate, deeply constricted at the septum, without guttules in each cell. **Asexual morph**: Undetermined.

##### Culture characteristics.

Ascospores germinating on PDA medium within 24 h and germ tubes produced from both ends. Colony on PDA medium reaching about 25 mm diam. After 30 days at room temperature in natural light, circular, with irregular margin, flattened, surface slightly rough, concentric. Colony from above white to pale yellowish, laterally becoming yellowish brown. Colony from below dark brown to black at the center, yellowish at the margins, slightly radiating with concentric rings.

##### Material examined.

China • Yunnan Province, Kunming, Kunming World Horticultural Expo Garden, 25°4'53.9328"N, 102°45'20.2536"E, on a diseased branch of *Olea europaea*, 15 July 2025, Wenli Li, WD 068 (HKAS 151735, holotype); ex-type, CGMCC3.29467, ex-isotype living culture, SWFCCC 250001.

##### Notes.

The BLASTn searches of the ITS sequence of *Melomastia kunmingensis* (CGMCC3.29467) resulted in 95% similarity (480/504 bp, 10 gaps) with *M. septata* (MFLUCC 22-0112) and 92% similarity with *Melomastia* sp. KH-2024a (GMB6242) (581/629, 13 gaps). The LSU BLASTn results showed 99% (851/856 bp, 1 gap) similarity with *M. italica* (MFLUCC 15-0160). The SSU and *tef1-α* BLASTn results showed 99% (938/939 bp, 0 gap) and 93% (837/901 bp, 2 gaps) similarity with *M. hydei* (MBSZU 25-003) and *M. septata* (Y50), respectively. In the phylogenetic analysis inferred from LSU-SSU-ITS-*tef1-α* sequences, *M. kunmingensis* is basal to *M. kevinhydei* with 83% ML and 1 BYPP support (Fig. 1). Comparing the LSU, SSU, ITS, and *tef1-α* sequences of *M. kunmingensis* and *M. kevinhydei* showed 0.2% (2/767 bp), 0.2% (2/822 bp), 6.2% (40/640 bp), and 5.8% (47/801 bp) nucleotide differences, respectively. These species share common morphological traits such as dark brown, obpyriform, semi-immersed to erumpent ascomata, 8-spored, bitunicate, cylindrical asci, and hyaline, uniseriate, and 2-septate ascospores. Ascospores of *M. kevinhydei* possess a large oil droplet per cell and are surrounded by a mucilaginous sheath when mature (Du et al. 2025), while *M. kunmingensis* lacks oil droplets during all developmental stages and does not form a mucilaginous sheath upon maturity.

#### Melomastia kevinhydei

Taxon classificationFungiSordariomycetes

H.Z. Du & Jian K. Liu, Mycosphere 16(2): 190 (2025)

7A7DF702-F5B7-58BB-9434-C17A048CA456

MycoBank No: 857555

Facesoffungi Number: FoF17825

Fig. 5

##### Description.

***Saprobic*** on diseased branches of *Olea europaea* L. **Sexual morph: *Ascomata*** visible as raised, dome-shaped, black dots on the host surface, solitary, scattered to gregarious. In vertical section, obpyriform, semi-immersed to erumpent, black, carbonaceous, ostiolate. ***Hamathecium*** 2–3 µm wide, numerous, filamentous, flexuose, branched, hyaline. ***Asci*** 96–145 × 6–14 µm (x̄ = 120 × 10 µm, n = 30), bitunicate, 8-spored, cylindrical, pedicellate, apically rounded, with minor ocular chamber. ***Ascospores*** 5–7 × 15–22 µm (x̄ = 6 × 18 µm, n = 30), uniseriate, hyaline, cylindrical, with rounded ends, 2-septate, constricted at the septum, with guttules in each cell, smooth-walled, surrounded by a mucilaginous sheath at maturity. **Asexual morph**: Undetermined.

**Figure 5.**
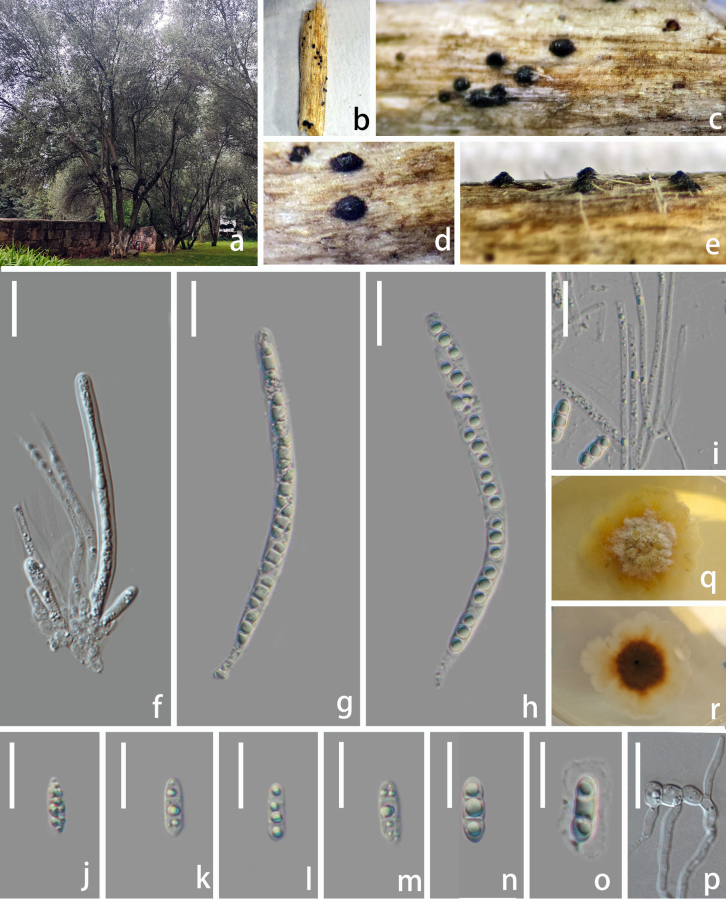
*Melomastia kevinhydei* (SWFC 250001). **a**. Habitat; **b–e**. Ascomata on the host; **f–h**. Asci; **i**. Hamathecium; **j–o**. Ascospores; **p**. Germinated ascospore; **q, r**. Culture (**q**. From above; **r**. From below). Scale bars: 20 μm (**f–p**).

##### Culture characteristics.

Ascospores germinating on PDA medium within 24 h and germ tubes produced from every cell. Colony on PDA medium reaching 25–30 mm diam. after 30 days at room temperature in natural light, circular to irregular, with dense white mycelium at the middle, sparser towards the edge; in reverse, yellowish brown at the middle, pale yellow at the entire margin.

##### Material examined.

China • Yunnan Province, Kunming, Kunming World Horticultural Expo Garden, 25°4'53.9328"N, 102°45'20.2536"E, on a diseased branch of *Olea europaea*, 15 July 2025, Wenli Li, WD 081 (SWFC 250001); living culture, SWFCCC 250002.

##### Notes.

*Melomastia kevinhydei* was introduced by Du et al. (2025) from *Nerium oleander* (Apocynaceae) in China. Our collections are morphologically identical to *M. kevinhydei*, and phylogeny also reveals a close relationship between them with high support. We report it as a new host record from *Olea europaea* in China.

#### Oedohysterium oleae

Taxon classificationFungiHysterialesHysteriaceae

W.L. Li, & C.L. Zhao sp. nov.

DC194860-0CEB-5CD3-A9EC-E744E0C8071C

MycoBank No: 862012

Facesoffungi Number: FoF19141

Fig. 6

##### Etymology.

The specific epithet refers to the host genus name, *Olea*.

**Figure 6.**
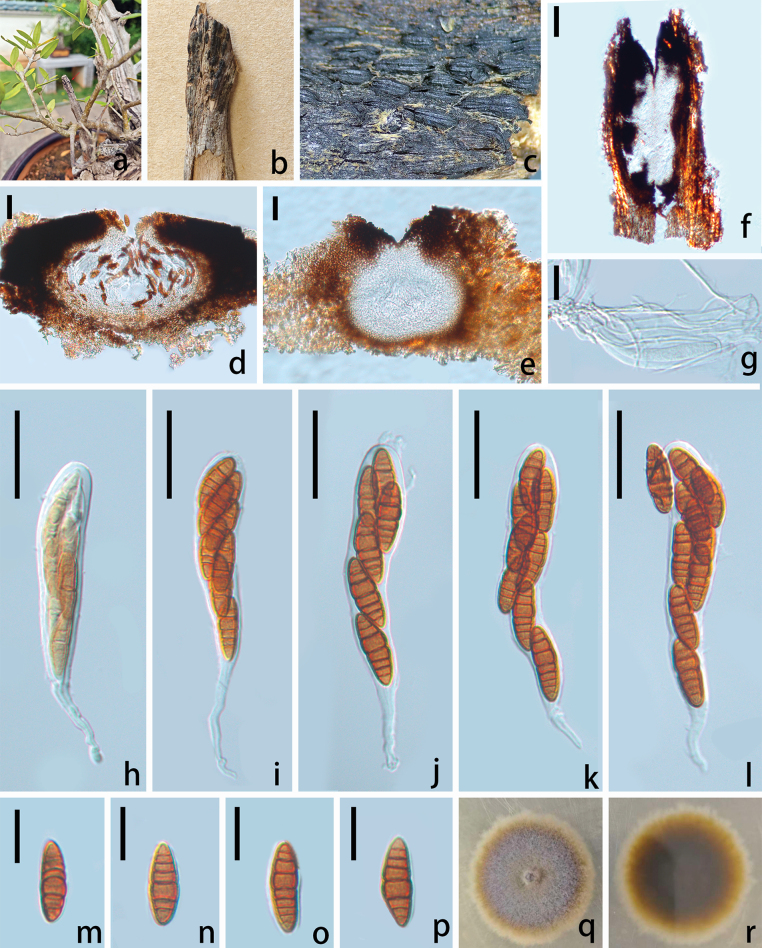
*Oedohysterium oleae* sp. nov., (HKAS 151736). **a**. Habitat; **b, c**. Ascomata on the host; **d, e**. Vertical section of ascoma; **f**. Transverse section of ascoma; **g**. Hamathecium; **h–l**. Asci; **m–p**. Ascospores; **q, r**. Culture (**q**. From above; **r**. From below). Scale bars: 50 μm (**d–f**); 40 μm (**g–l**); 20 μm (**m–p**).

##### Holotype.

HKAS 151736.

##### Description.

***Saprobic*** on diseased branches of *Olea europaea* L. ***Sexual morph*: *Hysterothecia*** scattered to subgregarious, dark brown, linear, often parallel but non-confluent laterally, sometimes lying at irregular angles, sessile on the substrate, surface usually longitudinally striate in age, in vertical section 260–350 µm high × 290–580 µm diam. (x̄ = 305 × 435 µm, n = 10), depressed globose, ostiolate. ***Ostiolar*** 70–100 µm diam. (n = 5), neck central, cylindrical. ***Peridium*** 380–850 µm wide (x̄= 615 µm, n = 10), comprising dense, several layers of brown to dark brown, compressed cells of ***textura angularis*** to ***textura prismatica***. ***Hamathecium*** 2–3 μm wide, hyaline, cellular, unbranched. ***Asci*** 120–160 × 20–24 µm (x̄ = 140 × 22 µm, n = 30), clavate, bitunicate, fissitunicate, 8-spored, long-stipitate, ascospores biseriate to subseriate in ascus, with a shallow ocular chamber. ***Ascospores*** 28–36 × 9–13 µm (x̄ = 32 × 11 µm, n = 30), broadly fusiform, straight or slightly curved, phragmospores, at first hyaline, then pale-yellow to brown, 6–8 septa (mostly 8), constricted at the central septum, with a prominent swollen or tumid supra-median cell, usually located just above the median septum. ***Asexual morph***: Not observed.

##### Culture characteristics.

Ascospores germinating on PDA medium within 24 h. Colony on PDA medium reaching 25 mm diam. After 30 days at room temperature, circular, flattened, surface slightly rough, concentric. Colony from above white to pale yellowish, laterally becoming yellowish brown. Colony from below, dark brown to black at the center, yellowish at the margins, slightly radiating with concentric rings.

##### Material examined.

China • Yunnan Province, Kunming, Kunming World Horticultural Expo Garden, 25°4'53.9328"N, 102°45'20.2536"E, on a diseased branch of *Olea europaea*. 12 April 2025, Wenli Li, WL 021 (HKAS 151736, holotype), ex-type, CGMCC3.29466, ex-isotype living culture, SWFCCC 250003.

##### Note.

*Oedohysterium oleae* is introduced as a new species based on its distinct morphology and phylogenetic results of a combined LSU-SSU-*tef1-α*-ITS dataset. Morphologically, *O. oleae* shares characters with the other three species in *Oedohysterium*, including phragmospores with a prominent, swollen supra-median cell. *O. oleae* differs from *O. sinense* by smaller (28–36 × 9–13 µm vs. 23–28 × 7–10 µm), smooth ascospores that become deep brown at maturity, as well as long-stipitate asci, and from *O. pulchrum* by the absence of longitudinal septa in the ascospores. Ascospores of *O. oleae* have a rounded apex, while those of *O. insidens* are pointed. Besides, Hysterothecia of *O. insidens* are comparatively smaller than *O. oleae* (50–250 × 200–500 µm vs. 260–350 × 290–580 µm), and striated laterally with age. In the phylogenetic analysis, *O. oleae* formed a sister group to *O. sinense* (CBS 123345) with strong statistical support (100% ML, 1 BYPP, Fig. 2), while another strain of *O. sinense* (EB 0339) formed a close relationship with two strains of *O. insidens* with 97% ML/1.00 BYPP support. A comparison of the 844 nucleotides across LSU reveals only 3 bp (0.3%) differences between *O. oleae* and *O. sinense*, however, SSU and *tef1-α* reveal 18/953 (1.8%) and 26/671 bp (3.8%) differences, respectively.

**Figure 7.**
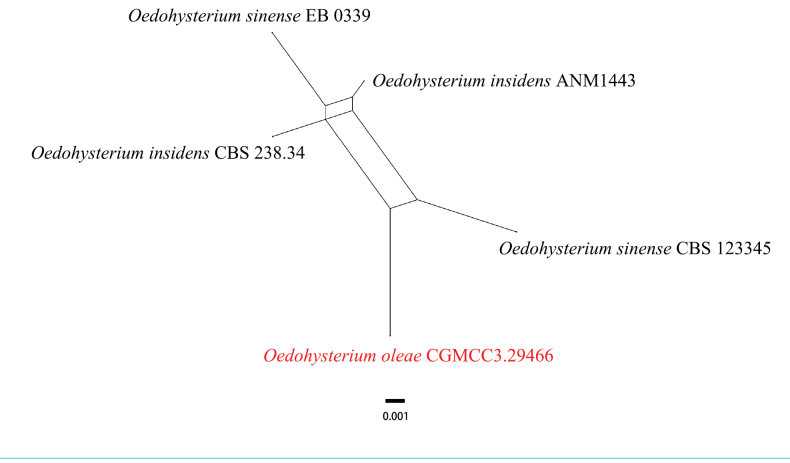
The splits graph from the pairwise homoplasy index (PHI) test was generated from the concatenated LSU-SSU-*tef1-α* sequence data of closely related species using splits decomposition. PHI test results (Φw) < 0.05 indicate significant recombination within the dataset.

Based on current evidence, phylogenetic results from the concatenated gene loci do not sufficiently delineate species boundaries within the genus *Oedohysterium*. Therefore, to further assess genealogical concordance and clarify species limits, we applied the Genealogical Concordance Phylogenetic Species Recognition (GCPSR) concept to the *Oedohysterium* clade. Analysis under a genealogical correlation model between neighboring strains of the clade showed a pairwise homoplasy index of Φw = 0.1353 (p > 0.05) (Fig. 7), indicating no significant recombination. The phylogenetic lineages and the evidence of genealogical discordance collectively suggest that *O. oleae* should currently be treated as a species distinct from *O. insidens* and *O. sinense*.

#### Parapyrenochaeta protearum

Taxon classificationFungiPleosporalesParapyrenochaetaceae

(Crous) Valenz.-Lopez, Crous, Stchigel, Guarro & Cano, Stud. Mycol. 90: 64 (2017)

F5F4329B-08A2-5792-95AE-732654DA56EF

MycoBank No: 820320

Facesoffungi Number: FoF08324

Fig. 8

##### Description.

***Saprobic*** on diseased branches of Nandina domestica Thunb. ***Sexual morph*: *Ascomata*** 130–285 μm high × 100–230 μm diam. (x̄ = 207 × 165 µm, n = 10), scattered, dark brown to black, immersed, globose to subglobose, uni-locular, papilla slightly erumpent through the host surface. ***Peridium*** 16–32 µm wide (x̄= 24 µm, n = 10), comprising brown, gradually paler toward the interior compressed cells of ***textura angularis*** to ***textura prismatica***. ***Hamathecium*** 2–3 μm wide, hyaline, cellular, unbranched. ***Asci*** 120–160 × 20–24 µm (x̄ = 140 × 22 µm, n = 30), clavate, bitunicate, fissitunicate, 8-spored, long-stipitate, ascospores biseriate to subseriate in ascus, with a shallow ocular chamber. ***Ascospores*** 28–36 × 9–13 µm (x̄ = 32 × 11 µm, n = 30), broadly fusiform, straight or slightly curved, phragmospores, at first hyaline, then pale-yellow to brown, 6–8 septa, constricted at the central septum, with a prominent swollen or tumid supra-median cell, usually located just above the median septum. ***Asexual morph***: Not observed.

**Figure 8.**
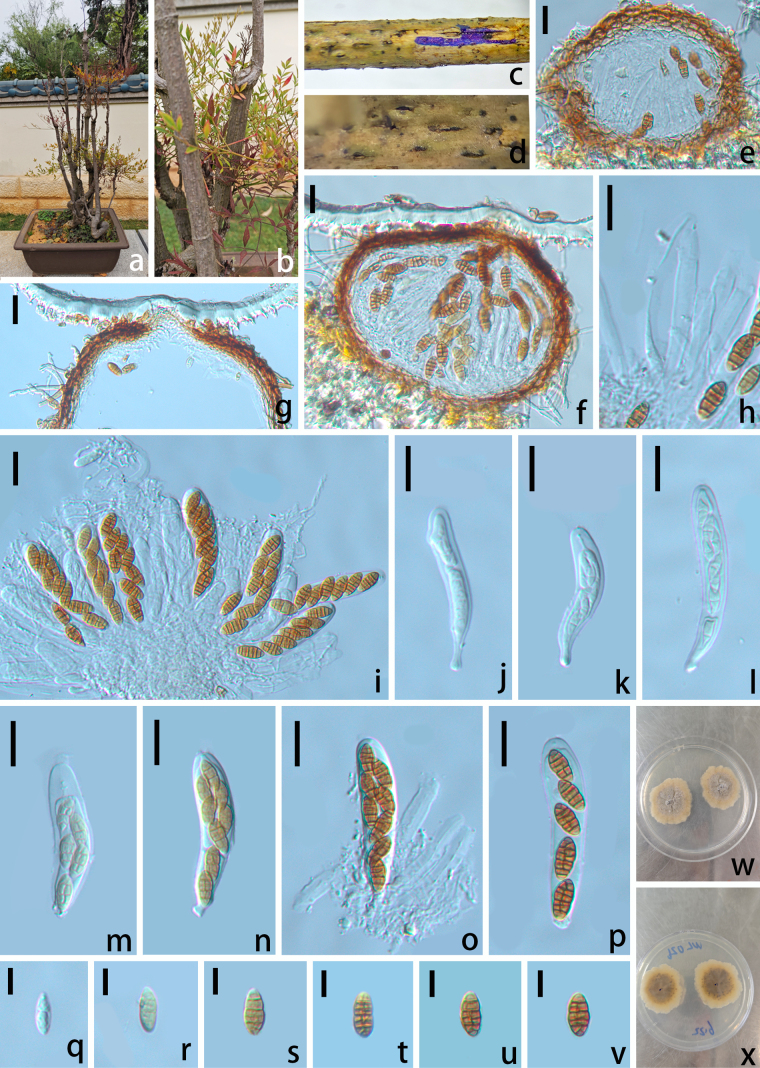
*Parapyrenochaeta protearum* (SWFC 250002). **a, b**. Habitat; **c, d**. Ascomata on the host; **e, f**. Vertical section of ascoma; **g**. Ostiole; **h**. Hamathecium; **i–p**. Asci; **q–v**. Ascospores; **w, x**. Culture (**w**. From above; **x**. From below). Scale bars: 50 μm (**e–p**); 20 μm (**q–v**).

##### Culture characteristics.

Ascospores germinating on PDA medium within 24 h. Colonies on PDA medium reaching 30 mm diam. after 30 days at 28 °C, circular, with irregular margin, flattened, surface slightly rough. Colony from above yellowish, laterally becoming yellowish brown at the center; in reverse pale brown at the middle, yellowish at the entire margin.

##### Material examined.

China • Yunnan Province, Kunming, Kunming World Horticultural Expo Garden, 25°4'53.9328"N, 102°45'20.2536"E, on a diseased branch of Nandina domestica. 12 April 2025, Wenli Li, WD 026 (SWFC 250002); living culture, SWFCCC 250004.

##### Note.

Phylogenetic analysis based on combined SSU, LSU, ITS, *rpb2*, *tef1-α*, and *tub2* sequences revealed that our isolate formed a well-supported subclade (100% ML, 1 BYPP; Fig. 3) with two strains of *Parapyrenochaeta protearum*. This species was originally described as *Pyrenochaeta protearum* by Crous et al. (2011). Subsequent multi-locus phylogenetic studies by Valenzuela-Lopez et al. (2018) demonstrated that *Pyrenochaeta protearum* was phylogenetically distinct from *Pyrenochaeta* species, leading to its reclassification into the new genus *Parapyrenochaeta*. Furthermore, the ex-type strain of *Pyrenochaeta pinicola* was found to be morphologically and genetically similar to *Pa. protearum*, resulting in the synonymization of *Py. pinicola* under *Pa. protearum* and its transfer to the genus *Parapyrenochaeta*.

The genus *Parapyrenochaeta* is characterized by a phoma-like asexual morph, whereas the sexual morph has been rarely observed. In this study, we report and describe the sexual morph of *Pa. protearum* for the first time.

## Discussion

The family Pleurotremataceae was established to accommodate the monotypic genus *Pleurotrema* (Watson 1929). Subsequently, the genera *Dyfrolomyces* (Pang et al. 2013) and *Melomastia* (Saccardo 1875) were classified within this family. As an increasing number of new species are introduced into Pleurotremataceae, *Dyfrolomyces* was synonymized under *Melomastia* based on overlapping morphological characteristics and multi-locus phylogenetic evidence (Li et al. 2022; Du et al. 2025). However, the generic boundaries within Pleurotremataceae remain ambiguous, with some species exhibiting transitional morphological traits (eg. *M. tiomanensis* and *M. chromolaenae*) that challenge the current classification (Kularathnage et al. 2023). *M. kunmingensis*, introduced in this study, displays distinct morphological features that diverge significantly from those of its close relatives, supporting its recognition as a new species based on both phylogenetic and morphological evidence.

The genus *Melomastia* demonstrates a notable and recurrent association with *Olea europaea*. Several species, including *M. winteri*, *M. sichuanensis*, *M. oleae*, and *M. fusispora*, have been found on *O. europaea* (Li et al. 2022). The discovery of *M. kunmingensis* on *O. europaea* in this study further strengthens evidence for a specific ecological link. Additionally, we report *M. kevinhydei* on *O. europaea* for the first time; it was originally described from *Nerium oleander* (Du et al. 2025), suggesting a potentially broader host range.

Olive, an ancient cultivated species introduced to China nearly a century ago, presents a compelling model for studying geographic differentiation and adaptation. The distribution and evolution of closely associated fungi, such as *Melomastia* may be influenced by the host’s introduction history and environmental adaptation. Investigating the genetic differentiation of *Melomastia* populations on olive in China can therefore yield valuable insights into the co-evolution and geographic diversification of host-associated mycobiota.

The genus *Oedohysterium* was introduced to accommodate two species transferred from *Hysterium* (*O. insidens* and *O. sinense*) and one from *Hysterographium* (*O. pulchrum*) (Boehm et al. 2009). No new species have been added since. Our proposed new species, *O. oleae*, differs morphologically from its congeners in ascospores, asci, and stipe dimensions. Mature ascospores of *O. oleae* predominantly have eight septa, whereas *O. insidens* and *O. sinense* are reported to have 6–9 septa.

Our phylogenetic analysis places *O. oleae* within the *Oedohysterium* clade, but the relationships between *O. insidens*, *O. sinense*, and *O. oleae* remain unresolved, consistent with previous studies (Boehm et al. 2009). A pairwise homoplasy index (PHI) test of the combined LSU, SSU, and *tef1-α* dataset showed no significant recombination among these three taxa, indicating they are not conspecific (Fig. 6). While the phylogenetic positions of some strains named *O. insidens* and *O. sinense* may require future re-evaluation, due to the lack of type specimen examinations and ITS sequences in public databases, we refrain from proposing taxonomic revisions here. The combined phylogenetic and morphological evidence robustly supports the description of our strain as the new species *O. oleae*.

The genus *Parapyrenochaeta* was introduced to accommodate *P. acaciae* and *P. protearum* (Valenzuela-Lopez et al. 2018). Accurate identification within this genus and its relatives is challenging, as many species share a phoma-like asexual morph, and the absence of a sexual stage complicates morphological differentiation (Valenzuela-Lopez et al. 2018; Li et al. 2020), making molecular phylogeny essential.

A key finding of this study is the first discovery of the sexual morph for the type species, *Parapyrenochaeta protearum*. This morph is characterized by muriform, yellow ascospores and clavate asci, features similar to those observed in the sexual morphs of Cucurbitariaceae (Jaklitsch et al. 2018). This discovery provides crucial morphological data that complements the molecular phylogenetic placement of the genus, leading to a more comprehensive taxonomic understanding of *Parapyrenochaeta*.

## Conclusion

We carried out fungal diversity investigations at the Kunming World Horticultural Expo Garden in Yunnan Province, a unique man-made ecosystem. Four fungal strains were isolated from the diseased branches of two cultivated plants, *Olea europaea* and Nandina domestica. Through integrated morphological characterization and multi-locus phylogenetic analyses, we identified two novel species: *Oedohysterium oleae* and *Melomastia kunmingensis*, which are phylogenetically and morphologically distinct from known species. Furthermore, we report *M. kevinhydei* as a new host record on *O. europaea*, indicating a potentially broader host range for this species. Additionally, we document, for the first time, the sexual morph of the type species, *Parapyrenochaeta protearum*, providing crucial morphological insights for a genus previously known only in its asexual state. These findings highlight the garden’s significance as a valuable reservoir of fungal diversity and underscore the need for further mycological explorations in similar and unique horticultural settings.

## Supplementary Material

XML Treatment for Melomastia kunmingensis

XML Treatment for Melomastia kevinhydei

XML Treatment for Oedohysterium oleae

XML Treatment for Parapyrenochaeta protearum

## Additional information

### Artificial Intelligence (AI) use

The authors accept full responsibility for the content of the manuscript, including the disclosure of any use of AI.

No AI tools were used in the preparation of this manuscript.

### Author ORCIDs

W. Li https://orcid.org/0000-0003-0370-8154

R. Jeewon https://orcid.org/0000-0002-8563-957X

G. Zhang https://orcid.org/0009-0006-6565-7072

H. Luo https://orcid.org/0009-0001-4550-8903

L. Zhao https://orcid.org/0009-0003-2553-982X

Z. Wang https://orcid.org/0000-0001-9022-3635

C. Zhao https://orcid.org/0000-0002-8668-1075

H. Zhou https://orcid.org/0000-0002-0724-5815

### Data availability

All of the data that support the findings of this study are available in the main text or Supplementary Information.
